# COPING WITH PERSISTENT NURSING HOME CHALLENGES: TEAM FUNCTIONING STRATEGIES TO EMPOWER DIRECT CARE WORKERS

**DOI:** 10.1093/geroni/igae098.3831

**Published:** 2024-12-31

**Authors:** Alfred Boakye, Jennifer Morgan

**Affiliations:** University of Maryland, Baltimore, Baltimore, Maryland, United States; Georgia State University, Atlanta, Georgia, United States

## Abstract

Nursing homes continue to experience staffing shortages, heavy workloads, poor quality indicators, and infection control challenges. Increasing levels of acuity and numbers of residents with challenging behaviors requires high-functioning teams in order for nursing homes to deliver high-quality person-centered care. Extant literature has described the benefits of developing and building teams to reduce staff conflict, reduce staff turnover and absenteeism, improve financial outcomes, and increase quality of care, motivation, and job satisfaction. Team functioning and improved communication can play a crucial role in delivering positive resident care outcomes. The aim of this study was to identify strategies that nursing homes can use to improve team functioning and communication in service of improved resident outcomes. Using a phenomenological qualitative approach with semi-structured interviews, twenty-five (25) nursing assistants were recruited from two high-quality nursing centers to understand team-related coping strategies adopted in order to combat persistent stressors. Interviews lasted 30-45 minutes. Interviewees were predominately Black (23), women (23), and had modal age range of 41-50 years old. Results of the analysis suggest that strategies such as formalizing team structures, dismantling cliques, encouraging the use of collaboration strategies, and tailoring check-in options for individual workers within teams (e.g., emails, phone calls and daily huddles) are critical to maximizing team functioning. These themes support our recommendation that nursing home leaders could profitably implement trauma-informed approaches to build more resilient teams. Trauma-informed principles such as safety, choice, collaboration, trustworthiness and empowerment offer values that can be infused into positive work cultures and support high-functioning teams.

